# Perinatal systemic gene delivery using adeno-associated viral vectors

**DOI:** 10.3389/fnmol.2014.00089

**Published:** 2014-11-14

**Authors:** Rajvinder Karda, Suzanne M. K. Buckley, Citra N. Mattar, Joanne Ng, Giulia Massaro, Michael P. Hughes, Manju A. Kurian, Julien Baruteau, Paul Gissen, Jerry K. Y. Chan, Chiara Bacchelli, Simon N. Waddington, Ahad A. Rahim

**Affiliations:** ^1^Gene Transfer Technology Group, UCL EGA Institute for Women’s Health, University College LondonLondon, UK; ^2^Experimental Fetal Medicine Group, Department of Obstetrics and Gynaecology, National University of SingaporeSingapore, Singapore; ^3^Department of Pharmacology, UCL School of Pharmacy, University College LondonLondon, UK; ^4^Neurosciences Unit, UCL Institute of Child Health, University College LondonLondon, UK; ^5^Clinical and Molecular Genetics Unit, UCL Institute of Child Health, University College LondonLondon, UK; ^6^Centre for Translational Research – GOSgene, UCL Institute of Child Health, University College LondonLondon, UK; ^7^School of Pathology, University of the WitwatersrandJohannesburg, South Africa

**Keywords:** adeno-associated virus, systemic delivery, perinatal, gene therapy, neurodegenerative disease, mouse models, non-human primates, metabolic disease

## Abstract

Neurodegenerative monogenic diseases often affect tissues and organs beyond the nervous system. An effective treatment would require a systemic approach. The intravenous administration of novel therapies is ideal but is hampered by the inability of such drugs to cross the blood–brain barrier (BBB) and precludes efficacy in the central nervous system. A number of these early lethal intractable diseases also present devastating irreversible pathology at birth or soon after. Therefore, any therapy would ideally be administered during the perinatal period to prevent, stop, or ameliorate disease progression. The concept of perinatal gene therapy has moved a step further toward being a feasible approach to treating such disorders. This has primarily been driven by the recent discoveries that particular serotypes of adeno-associated virus (AAV) gene delivery vectors have the ability to cross the BBB following intravenous administration. Furthermore, safety has been demonstrated after perinatal administration mice and non-human primates. This review focuses on the progress made in using AAV to achieve systemic transduction and what this means for developing perinatal gene therapy for early lethal neurodegenerative diseases.

## INTRODUCTION

The development of intravenously administered therapies for neurodegenerative diseases is often impeded by the blood–brain barrier (BBB). The BBB isolates and protects the central nervous system (CNS) by restricting large or hydrophobic molecules such as viruses and large proteins from crossing into the CNS (Rubin and Staddon, 1999). Remarkable efficacy has been reported in clinical trials for neurodegenerative disorders using *ex vivo* hematopoietic stem cell gene therapy (Cartier et al., 2009; Biffi et al., 2013). While this approach is hugely promising for those diseases where secretion of a soluble protein from bone marrow derived microglia and therapeutic recapture from neighboring neural cells is possible, other conditions may not be amenable to this approach. Therefore, highly efficient and direct delivery of genes, ideally targeted to specific neural cells using cell type specific promoters, would be required. The adeno-associated virus (AAV) vector system has been subject to recent, intense, scrutiny due to: (1) high efficiency of neural cell transduction (Gray et al., 2011); (2) excellent safety profile in humans (Nakai et al., 2005); (3) low toxicity (Daya and Berns, 2008); and (4) lack of integration into the host genome, therefore imposing a lower risk of insertional oncogenesis associated with integrating vectors such as lentivirus (Themis et al., 2005; Howe et al., 2008). The post-mitotic status of neurons makes them a good target for non-integrating vectors. Thus, AAV is now a commonly used vector for clinical trials addressing neurological disease. This must be balanced with specific disadvantages associated with AAV vectors: (1) a limited packaging capacity of around 4.7 kb (Dong et al., 2010) and (2) the possibility of pre-existing humoral response to capsid protein because of prior wild-type AAV infections (Boutin et al., 2010).

Numerous AAV serotypes have been isolated from human and non-human primates (NHPs). Their tropisms following intravenous administration have been studied in mice and NHPs, these include AAV6, 6.2, 7, 8, 9, rh.8, rh.10, rh.39, and rh.43 (Towne et al., 2008; Zincarelli et al., 2008; Gray et al., 2011; Yang et al., 2014). Interestingly, AAV9 has been shown to cross the BBB in adult and neonatal animals following intravenous infusion of the virus (Zincarelli et al., 2008; Duque et al., 2009; Foust et al., 2009; Fu et al., 2011). Other AAV serotypes have since demonstrated the ability to cross the BBB in neonatal mice following intravenous administration with AAVrh.10 and AAVrh.8 both showing promise (Zhang et al., 2011).

An example of a lethal neurodegenerative monogenic disease with visceral involvement is neuronopathic Gaucher disease (GD). Patients exhibit hepatomegaly and splenomegaly in addition to the ultimately lethal brain pathology (Gupta et al., 2011). Enzyme replacement therapy (ERT) has been highly successful in treating the visceral pathology associated to GD (Cox, 2013) and other lysosomal storage disorders (Ortolano et al., 2014). However, the presence of the BBB has severely restricted recombinant enzyme from crossing into the CNS and rendering ERT ineffective in preventing death from neurodegeneration (Pastores et al., 1993; Wynn et al., 2009). Such systemic diseases will ideally require a systemic treatment. AAV serotypes that can be intravenously administered and cross the BBB raises hope that this approach may have clinical potential for treating such disorders. Furthermore, the prospect of administering gene therapy during the perinatal period is attractive for a number of reasons. Here we discuss the potential of AAV administered during the perinatal period to treat systemic disorders, with an emphasis upon neurodegenerative conditions, using a selection of studies from a rapidly growing field to highlight progress.

## PERINATAL GENE DELIVERY

Gene delivery to the developing fetus or neonate has been discussed extensively over the years (David and Peebles, 2008; Rahim et al., 2010; Buckley et al., 2011; Coutelle and Waddington, 2012; Mattar et al., 2012; Roybal et al., 2012). While this is yet to be realized in the clinic, the advantages that such an approach offers are appealing (1) A higher vector to cell ratio enhancing the efficiency of gene delivery, (2) A naïve immune system allows for the induction of ‘tolerance’ to vector-mediated expression of a protein that the body may not recognize as self, (3) Reduced chances of an immune response to a viral vector since the fetus is unlikely to have had previous viral infections, (4) Potentially enhanced access of vectors to progenitor and stem cell niches, and (5) The ability to prevent pathology from manifesting – arguably most important in the context of acute neurodegenerative disorders where susceptible post-mitotic neurons must be protected at the earliest possible moment to prevent irrevocable loss. The arguments in favor of *in utero* gene therapy must be balanced with those against such as (1) Is there an established method for early diagnosis for the disease in question? (2) If the fetus is diagnosed with an intractable genetic disorder then would the mother opt to terminate the pregnancy? We refer the reader to a separate elegant review by Coutelle and Ashcroft (2012) that discusses the ethical, legal and societal implications. (3) Fetal developmental and cell physiological abnormalities caused by overexpression of the therapeutic transgene. Realistically, gene therapy for the neonate will be developed first. Symptoms may be more obvious; further investigation by newborn screening programs or scans may facilitate and confirm diagnosis followed by administration of gene therapy – but in many cases of acute and aggressive neurodegenerative disorders that present during gestation, neonatal intervention could already be too late.

## AAV IN FETAL AND NEONATAL RODENTS

Very few studies have examined systemic gene delivery via intravenous administration to the fetal rodent. This is probably due to the technical expertise required to perform this procedure. Rahim et al. (2011) described extensive reporter gene expression in mice following *in utero* intravenous injection of AAV9. This vector efficiently crossed the BBB and mediated global gene expression throughout the nervous system. More studies have examined gene delivery following intravenous administration of AAV into neonatal rodents. Foust et al. (2009) showed that intravenous injections of AAV9 carrying the GFP reporter gene into neonatal mice resulted in extensive transduction of neuronal cells within the spinal cord and the brain and within the heart and skeletal muscles. Similarly, Duque et al. (2009) showed efficient transduction of motor neurons within the spinal cords of adult mice and cats after intravenous injections of AAV9. Foust et al. (2009) further elaborated on their previous AAV9 study by rescuing an early lethal mouse model of spinal muscular atrophy (SMA). Remarkably, a single intravenous injection at birth of AAV9 carrying the *SMN* gene was sufficient to correct the neuromuscular electrophysiology and motor functions (Foust et al., 2010). In contrast, injection of therapeutic AAV9 vector at later time points (5 and 10 days after birth) produced partial correction or little effect, respectively. This highlights the critical importance of identifying the therapeutic window of opportunity that may be narrow and early in development. Towne et al. (2008) demonstrated motor neuronal transduction within the spinal cord and the brain stem through intravenous administration of AAV6 to adult mice. AAV8, 9, and rh.10 also achieved impressive nervous system transduction (Yang et al., 2014). This also supports the observations of impressive neuronal and glial transduction in a separate study using AAV9 in adult mice (Gray et al., 2011). Furthermore, a systematic comparison of various AAV serotypes in the neonatal mouse revealed that AAV9 was highly efficient in crossing the BBB, although AAVrh.10 also holds great promise and must be investigated further (Zhang et al., 2011). To date, given the larger body of evidence and data on systemic delivery, the review will focus on AAV9.

The timing of systemic administration of AAV9 is also important in the context of gene expression in particular neural cells. Foust et al. (2009) demonstrated that AAV9 carrying the GFP gene driven by the chicken beta actin hybrid promoter effectively transduces neurons when intravenously administered to neonatal mice. However, expression becomes preferentially astrocyte specific when administered to adult mice. Further analysis revealed that this ‘switch’ between expression in cell types occurs within the first 10 days of life (Foust et al., 2010). Rahim et al. (2011) reported that intravenous injection of AAV9 carrying the GFP gene driven by the CMV promoter to E15 fetal mice led to preferential neuronal expression and minimal astrocyte expression. When the same vector was administered to P1 neonatal mice, preferential astrocyte expression was observed. In some cases, expression throughout the nervous system may not be required and preferably targeted to a specific neuronal population. This refinement in targeted gene expression will require more work in identifying suitable promoters or microRNA sequences.

## AAV IN FETAL AND NEONATAL NON-HUMAN PRIMATES

Non-human primates are a valuable pre-clinical model given the significant difference between fetal and neonatal development and cell type expression in rodents and humans. Mattar et al. (2013) evaluated the ability of AAV9 to transduce the CNS through a single intravenous injection into a late gestation fetal macaque. The administration of the virus was conducted using an established ultrasound guided clinical protocol that is routinely used worldwide. Examination of administered animals at 6 and 14 days of age revealed significant neuron transduction in the CNS and the PNS, with a lower efficiency seen in astrocytes. Furthermore, significant transduction in the skeletal muscle, heart and liver was observed. This work supports the findings of Rahim et al. (2011) in fetal mice intravenously administered with AAV9. However, the potential differences between mouse and NHPs were highlighted by Bevan et al. (2011) who intravenously administered AAV9 carrying the GFP gene driven by the chicken beta actin hybrid promoter to NHPs at P1, P30, and P90. Monkeys that received vector at P1 showed mostly astrocyte and microglial targeting in the brain that is in contrast to mice that received the same vector at P1 and demonstrated predominantly neuronal transduction (Foust et al., 2010). Bevan et al. (2011) attribute this discrepancy to differences in the timing of gliogenesis between the two species. The same study also reported sustained expression in the musculature and liver of injected monkeys. **Table 1** summarizes the key features described above. Intravenous injection of the vector used by Bevan et al. (2011) has also been administered to older 3–4 year old rhesus macaques (Bevan et al., 2011; Gray et al., 2011). 4-weeks after injection, analysis showed similar gene expression profiles – again predominantly glial in the brain with extensive transduction of the musculature and liver. Interestingly, monkeys that were serotype positive for AAV9 and had pre-existing circulating AAV9 neutralizing antibodies exhibited significantly reduced levels of transduction (Gray et al., 2011). This issue will be further discussed later but represents a strong argument for administering during the perinatal period where the subject is less likely to have been exposed to AAV infection and more likely to be serotype negative.

**Table 1:** Summary of intravenously administered AAV9-mediated gene delivery studies in perinatal mice and non-human primates.

Species	Developmental age at administration	Promoters	Transduction pattern	Reference
Mouse	E15	Cytomegalovirus (CMV)	Global transduction of central and peripheral nervous system – preferential neuronal transduction. Visceral transduction.	Rahim et al. (2011)
Mouse	PI	Chicken-β-actin hybrid promoter (CB) CMV	Wide spread delivery to central nervous system – preferential targeting of neurons or astrocytes. Heart and skeletal muscles.	Foust et al. (2009, 2010), Rahim et al. (2011)
NHP	Late gestation (0.9G)	CMV	Wide spread delivery to the central and peripheral nervous system – preferential neuronal transduction. Liver, heart, and skeletal muscles.	Mattar et al. (2013)
NHP	PI	CB	Preferential glial transduction in the brain and neurons in dorsal root ganglia and motor neurons of spinal cord. Muscle, liver, Kidney, spleen, heart, lungs, adrenal medulla, intestines, testis.	Bevan et al. (2011)

## CLINICAL TRANSLATION OF SYSTEMIC AAV-MEDIATED GENE THERAPY

Systemic delivery of AAV vectors in patients has already been demonstrated in a recent successful clinical trial using AAV8 to express the FIX gene to treat patients with the blood clotting disorder hemophilia B (Nathwani et al., 2011). AAV8 carrying the FIX gene driven by the LP1 liver specific promoter was administered, intravenously, to six patients with severe hemophilia B all over the age of 18 years old. Four of the six patients did not require prophylactic recombinant FIX. The other two patients that received a higher dose of vector either developed an AAV8-capsid specific T cell response or a small increase in liver enzyme levels – both symptoms were treated using glucocorticoids (Nathwani et al., 2011). While this study is in older patients, it provides an important proof-of-principle; high doses of AAV can be intravenously administered and tolerated without any life-threatening adverse effects. Furthermore, a Phase I clinical trial for the neuromuscular condition SMA is currently recruiting patients less than 9 months old (ClinicalTrials.gov Identifier: NCT02122952). Patients will receive an intravenous infusion of AAV9 carrying the therapeutic SMN gene driven by the CMV enhancer/chicken-beta-actin promoter. Interestingly, an inclusion criteria is onset of disease at birth to 6 months of age and patients are eligible to be included if they are 9 months or younger at time of infusion. The young age of the potential participants exemplifies the volition to intervene as early as possible and vindicates a strategy to treat diseases that may manifest at birth. This trial will also address potential logistical and immunological barriers that have been proposed to impede such an approach. It has been suggested that this approach for using AAV9 to treat neurodegenerative diseases would require large vector preparations to achieve the necessary therapeutic transduction levels in the CNS (Foust et al., 2009; Gray et al., 2011). Producing the amount of vector required for a human trial could be challenging with the current AAV production technology, although the baculoviral system (Cecchini et al., 2011) has been successfully used by uniQure to manufacture the first licensed gene therapy product in Europe using an AAV vector, Glybera (Yla-Herttuala, 2012).

Administering such a high titre may result in an adverse immune response, particularly in those patients that have low levels of pre-existing AAV antibodies, reducing the efficiency of therapy and increasing the chance of an adverse immune response (Boutin et al., 2010; Samaranch et al., 2012). Treating younger and, therefore, smaller patients may well overcome any issue of production and also pre-existing antibodies against AAV9 (although an exclusion criteria in the SMA trial is the presence of anti-AAV9 antibodies at a titre of >1:50 as determined by ELISA binding immunoassay). However, high levels of maternal alloantibodies against AAV could trigger an immunization response through maternal microchimerism that is thought to play a role in the development of the fetal immune system (Merianos et al., 2009; Jeanty et al., 2014).

A criticism of perinatal gene therapy is diagnosing a fetus or neonate early enough for therapeutic intervention. This is dependent upon diagnostic technologies and resources in any given country, e.g., the availability of prenatal screening and diagnosis programs. Screening programs even for rare neurological conditions have been established in communities that have a high prevalence such as Tay–Sachs and GD in the Ashkenazi Jewish populations (Eng et al., 1997). Advances in ultrasound technology have led to the ability to identify fetal abnormalities *in utero*. An example of this is Niemann–Pick disease type C where although the severity of disease and age of onset can be highly variable, fetal presentation in the form of splenomegaly, hepatomegaly, and ascites are detectable by ultrasound scan (Spiegel et al., 2009). The continuing development of next generation sequencing (NGS) over the past decade may ultimately hold the key to early or pre-term diagnosis. The ability to obtain fetal genetic information during pregnancy from the mother’s blood has been a long-term goal. Originally, studies focused on identification of such cells in the maternal circulation. Once cell-free fetal DNA (cffDNA) was identified (Lo et al., 1997), the focus directed toward ways of analyzing such DNA material for prenatal testing. Recently, non-invasive prenatal diagnosis (NIPD) utilizing cffDNA as early as 7 weeks’ gestation has been introduced into clinical practice as reviewed by Daley et al. (2014). This approach has been successfully used for fetal gender identification (Devaney et al., 2011), a highly accurate screening test for aneuploidies like trisomy 21 (Fan et al., 2008), other major trisomies (Norton et al., 2012), sex chromosomes abnormalities (Bianchi et al., 2012; Futch et al., 2013), and the diagnosis of some single gene disorders including cystic fibrosis, thalassaemia, and achondroplasia (Lench et al., 2013).

A more reliable, sensitive and cost effective method for NIPD in monogenic disorders is provided by NGS platforms. Now available in routine diagnostic laboratories, they can accurately quantify certain sequences in the maternal blood. An example was for the identification of Down Syndrome by whole genome sequencing by quantifying cffDNA sequences mapping to chromosome 21 (Fan et al., 2008). A more recent application of NGS in NIPD for b-thalassemia has been described (Lam et al., 2012).

Next generation sequencing presents many advantages over standard PCR-based genetic testing. The high throughput of NGS allows samples from different patients to be multiplexed in a single run. This combined with the ability to design assays with multiple mutations in multiple genes to be included in a single test are allowing a reduced cost per sample as well as a quick turn-around-time for reporting results. With the cost of NGS constantly decreasing and the imminent implementation of whole exome sequencing (which allows the simultaneous sequence of all coding exons) in diagnostic laboratories, NGS and NIPD will be increasingly used for the diagnosis of a range of monogenic disorders (Lench et al., 2013). Although the cost is still high and the time of data analysis long, whole genome sequencing could be implemented to profile the entire fetal genomes (Lo et al., 2010).

## CONCLUDING REMARKS

Perinatal gene therapy has distinct advantages (Summarized in **Table 2**) and is essential in addressing those diseases that manifest during the neonatal stage or even *in utero*. This is particularly important in early lethal neurodegenerative disorders where vulnerable post-mitotic neurons must be rescued or risk irrecoverable loss. The presence of additional systemic pathology is a further complication. However, the potential of intravenously administered AAV to provide systemic gene therapy is highly attractive in treating such pleiotropic conditions. Of course, there are significant practical issues that need to be overcome for perinatal gene therapy to be realized in the clinic. However, we are moving in the right direction with advances in prenatal diagnostic technologies, clinical trials demonstrating that intravenously administered AAV vectors are safe and plans to take this further in young children with SMA. The ascendancy of gene therapy through a number of recent successful gene therapy trials (Gaspar et al., 2004; Bainbridge et al., 2008; Cartier et al., 2009; Nathwani et al., 2011; Aiuti et al., 2013; Biffi et al., 2013) and the approval of the first gene therapy product to be sold in Europe (Yla-Herttuala, 2012) has meant that the field in general is gathering momentum and is an opportune moment to bring systemic perinatal gene therapy into the limelight.

**Table 2:** Summary of using AAV in perinatal gene therapy and its clinical implication.

**AAV transduction efficiency**
• The ability of AAV serotypes to cross the BBB highlights its systemic transduction capabilities.
• Intravenous administration of AAV9 at either fetal or neonatal stage of development in rodents and NHP targets different cell types within the nervous system; neurons via fetal intravenous (iv) and astrocytes and microglia via neonatal iv administration.
• The iv approach is ideal for systemic neurodegenerative disorders.
**Advantages of perinatal gene delivery**
• Efficient gene delivery is achieved due to; large vector to cell ratio, induction of immune-tolerance (at least in rodents) to foreign protein expression.
• Ideal for early lethal neurodegenerative diseases as it allows for therapeutic transduction of cells and organs prior to disease pathology manifestation.
**Potential disadvantages of perinatal gene therapy**
• Lack of diagnostic technologies or infrastructure to identify disease in patients early enough.
• *In utero* ultrasound or exposed surgical procedure may impose risk such as fetal loss or preterm birth.
• Over expression of the therapeutic transgene in the fetus may cause developmental abnormalities.
• Fetal iv preferentially targets neurons and not astrocytes.
**Clinical implication**
• Perinatal gene therapy has been successful in a number of animal CNS diseases.
• Intravenous route of administration is favorable for its ability to cross the BBB and target other peripheral organs.
• Particular AAV serotypes ability to cross the BBB provides and alternative to intracranial administration which would require surgery and its attendant risks.
• Successful clinical trials have taken place where systemic delivery of AAV and disease specific transgene has been delivered topically and intravenously to patients.

## Conflict of Interest Statement

The authors declare that the research was conducted in the absence of any commercial or financial relationships that could be construed as a potential conflict of interest.
